# Targeting adipocyte differentiation with CRT0066101: activation of AMPK signaling in 3T3-L1 cells

**DOI:** 10.3389/fphar.2025.1645587

**Published:** 2025-08-14

**Authors:** Jinque Luo, Ling Wang, Li Zhang, Wenyu Tang, Meijing Deng, Yuxin Shi, Kun Xu, Qingjun Wu, Jieyang Zhao, Jinghuan Zhang, Xin Li

**Affiliations:** ^1^ Hunan Provincial Key Laboratory of the Research and Development of Novel Pharmaceutical Preparations, “The 14th Five-Year Plan” Application Characteristic Discipline of Hunan Province (Pharmaceutical Science), College of Pharmacy, Changsha Medical University, Changsha, Hunan, China; ^2^ Hunan Provincial University Key Laboratory of the Fundamental and Clinical Research on Functional Nucleic Acid, College of Pharmacy, Changsha Medical University, Changsha, Hunan, China; ^3^ The National and Local Joint Engineering Laboratory of Animal Peptide Drug Development, College of Life Science, Hunan Normal University, Changsha, Hunan, China

**Keywords:** obesity, adipocyte differentiation, CRT0066101, 3T3-L1, AMPK

## Abstract

**Introduction:**

Obesity is characterized by excessive fat accumulation resulting from adipocyte hypertrophy and hyperplasia, with adipocyte differentiation being a central process driving these changes.

**Methods:**

The anti-adipogenic effects of CRT0066101 (CRT), a pan-protein kinase D (PKD) inhibitor, were evaluated in 3T3-L1 adipocytes. Potential drug targets of CRT were predicted using network pharmacology analysis. The expression of adipocyte-specific genes and proteins was assessed by western blotting and qRT-PCR. To examine the involvement of the AMPK pathway, cells were co-treated with CRT and the AMPK inhibitor Compound C.

**Results:**

CRT significantly inhibited early-stage adipocyte differentiation, reduced lipid accumulation, and downregulated key adipogenic transcription factors, including PPARγ and C/EBPα. Mechanistically, CRT activated the AMPK pathway, a known negative regulator of adipocyte differentiation. Network pharmacology analysis further supported the involvement of AMPK in CRT’s anti-adipogenic action.

**Discussion:**

These findings identify CRT as a novel modulator of adipocyte differentiation through AMPK activation and highlight its potential as a therapeutic candidate for obesity and metabolic syndrome.

## Introduction

Obesity has emerged as a global epidemic and a huge public health concern, substantially contributing to the global burden of noncommunicable diseases, including cardiovascular disease, type 2 diabetes, and numerous cancers (Piché et al., 2020; Aron-Wisnewsky et al., 2021; Li et al., 2025; Huang et al., 2024a). The rising prevalence of obesity underscores the urgent need to comprehend its fundamental principles and to develop effective strategies that promote healthier lifestyles and improve clinical outcomes.

A hallmark of obesity is the excessive expansion of adipose tissue, which occurs through two mechanisms: Hypertrophy, the enlargement of existing adipocytes due to triglyceride accumulation, and hyperplasia, an increase in adipocyte number driven by adipogenesis—the differentiation of precursor cells into mature adipocytes (Ghaben and Scherer, 2019; Wang et al., 2020). Adipogenesis is a tightly controlled biological process that plays a central role in maintaining adipose tissue homeostasis and systemic energy balance. Under normal physiological conditions, it ensures adipose tissue plasticity and metabolic adaptability. However, in obesity, adipocyte differentiation becomes excessive or dysregulated, characterized by an abnormal increase in adipocyte number, impaired maturation, dysfunctional lipid storage, and altered adipokine secretion (Fang et al., 2024; Lyu et al., 2024). Consequently, the expanding adipose tissue struggles to fulfill the increasing lipid storage demands. Once this capacity is exceeded, newly formed or hypertrophied adipocytes begin to experience various forms of cellular stress, including endoplasmic reticulum (ER) stress, oxidative stress, and hypoxia (Horwitz and Birk, 2023; Kawai et al., 2021). These stressors contribute to the development of metabolically unhealthy adipose tissue, driving chronic inflammation, insulin resistance, and a vicious cycle of metabolic dysfunction (Longo et al., 2019; Ahmed et al., 2021). As a result, dysregulated adipogenesis not only compromises the protective role of adipose tissue but also promotes systemic metabolic disorders such as type 2 diabetes, dyslipidemia, and cardiovascular disease (Horwitz and Birk, 2023; Longo et al., 2019). Targeting adipocyte differentiation thus presents a promising strategy to limit adipose expansion and restore metabolic balance. Pharmacological inhibition of adipogenesis may reduce lipid accumulation and alleviate obesity-related complications.

Excessive or dysregulated adipogenesis drives adipose tissue dysfunction, insulin resistance, and chronic low-grade inflammation. This process is regulated by a complex interplay of extracellular cues, intracellular signaling pathways, and sequential activation of transcription factors (Patel et al., 2024; Zhao et al., 2024; Malibary, 2023). Adipogenic transcriptional regulators such as peroxisome proliferator-activated receptor gamma (PPARγ), CCAAT/enhancer-binding protein alpha (C/EBPα), fatty acid-binding protein 4 (FABP4), and adiponectin play a crucial role in this process (Zhao et al., 2024; Han et al., 2022). These transcription factors orchestrate transcriptional programs that govern lipid biosynthesis, energy metabolism, and adipocyte maturation (Zhao et al., 2024; Malibary, 2023). Consequently, pharmacological compounds or small molecules capable of modulating the expression or activity of PPARγ and C/EBP have the potential to alter adipogenic outcomes and represent promising targets for therapeutic intervention in obesity and related metabolic disorders.

A well-established cell culture model has significantly advanced our understanding of the molecular mechanisms governing terminal adipocyte differentiation. Among the various preadipocyte lines and differentiation protocols available, 3T3-L1 cells remain the most widely used model system (Zebisch et al., 2012; Zhou et al., 2025; Al-Sayegh et al., 2021). Adipocyte differentiation in 3T3-L1 cells is typically induced using a cocktail of isobutylmethylxanthine (IBMX), dexamethasone, and insulin, which mimics the hormonal environment necessary for adipogenesis. Cyclic AMP (cAMP) plays a central role in this process by activating cAMP-response element-binding protein (CREB) through phosphorylation. Activated CREB promotes the transcription of C/EBPβ, a key early adipogenic transcription factor (Mubtasim et al., 2023). IBMX, a non-specific phosphodiesterase inhibitor, elevates intracellular cAMP levels, thereby stimulating the CREB–C/EBPβ axis and promoting adipocyte differentiation (Schroeder et al., 2012; Choi et al., 2020). Dexamethasone, a synthetic glucocorticoid, contributes to adipogenesis by inducing C/EBPα expression, enhancing the transcriptional activity of C/EBPβ through acetylation, and upregulating C/EBPδ via glucocorticoid receptor activation (Ehrlund et al., 2017). Together, these factors initiate and coordinate the transcriptional cascade necessary for the terminal differentiation of preadipocytes into mature adipocytes.

Protein kinase D (PKD), a family of serine/threonine kinases comprising PKD1, PKD2, and PKD3, has emerged as a potential regulator of metabolic function (Wang, 2006). PKDs are involved in diverse cellular processes, including proliferation, angiogenesis, apoptosis, inflammation, and vesicular trafficking (Roy et al., 2017; Zhang et al., 2021; Yuan et al., 2022; Steinberg, 2021). Recent studies suggested that distinct PKD isoforms participate in the regulation of lipid metabolism and energy homeostasis, with growing evidence linking their activity to the development of obesity (Wit et al., 2024; Löffler et al., 2018; Trujillo-Viera et al., 2021). Given the unique metabolic roles of PKD isoforms, pharmacological modulation of PKD activity presents a promising strategy for metabolic disease intervention. For instance, the pan-PKD inhibitor CRT0066101 (CRT) is a potent and selective small-molecule inhibitor of PKD, originally developed for its anti-proliferative and anti-inflammatory properties in cancer and immune-related disorders, which has shown efficacy both *in vitro* and *in vivo* against various human malignancies (Harikumar et al., 2010; Li et al., 2018; Cui et al., 2023). CRT exhibits moderate lipophilicity, which enhances cellular uptake and promotes efficient engagement with PKDs. It binds to PKDs with high affinity and selectivity while demonstrating minimal off-target effects (Harikumar et al., 2010; Wang and Wipf, 2023). By inhibiting PKD activity, CRT downregulates critical downstream effectors such as NF-κB and NLRP3, contributing to its established anti-proliferative and anti-inflammatory actions (Harikumar et al., 2010; Cui et al., 2023). Although CRT has demonstrated promising preclinical efficacy in PKD-driven pathologies, its potential in non-oncological contexts remains largely unexplored. In particular, its role in adipocyte differentiation and metabolic regulation warrants further investigation.

In this study, we investigated the effects of CRT on adipogenesis using the well-established 3T3-L1 preadipocyte model. We assessed its impact on lipid accumulation and the expression of adipogenic markers, and we explored the underlying molecular mechanisms. This study aimed to uncover a previously unrecognized role of CRT in modulating adipocyte differentiation and to evaluate its potential as a therapeutic candidate for obesity and related metabolic disorders.

## Materials and methods

### Chemicals and reagents

**FIGURE 1:**
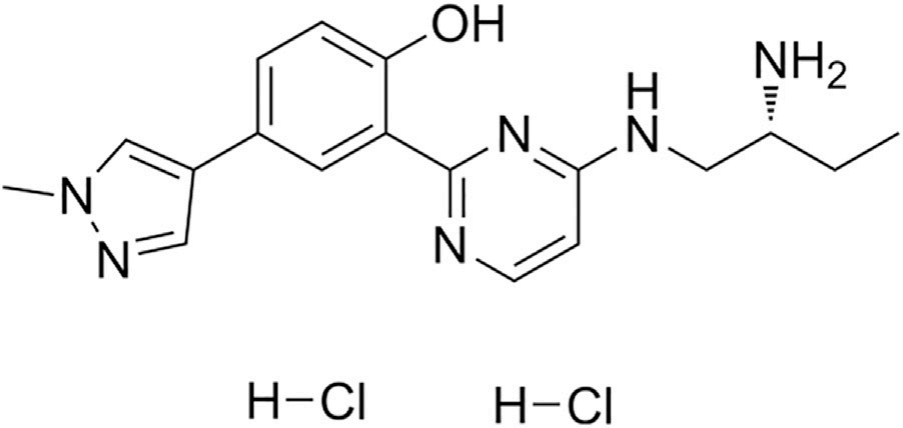
Chemical structures of CRT.

CRT0066101 (CRT) was purchased from Sigma-Aldrich Biotech Co. (St. Louis, MO, USA). The structure of CRT0066101 was shown in Figure 1. Compound C, 3-isobutyl-1-methylxanthine (IBMX), dexamethasone (Dex), and insulin were obtained from MedChemExpress Biotech Co. (Shanghai, China). Oil Red O was obtained from Solarbio Biotech Co. (Beijing, China). Dulbecco’s modified eagle medium (DMEM) was obtained from KeyGEN BioTECH Co. (Nanjing, China). Fetal bovine serum (FBS) was purchased from Gibco Biotech Co. (Waltham, MA, USA). TRIzol reagent for RNA extraction was obtained from Invitrogen Biotech Co. (Carlsbad, CA, USA). The BCA protein assay kit, RIPA lysis buffer, and protease and phosphatase inhibitors were purchased from Beyotime Biotech Co. (Shanghai, China). Antibodies against C/EBPα, PPARγ, Adiponectin, p-AMPK, and AMPK were purchased from Diagbio Biotech Co. (Hangzhou, China). Antibodies against p-ACC, ACC, PKD1, and GAPDH were purchased from HuaBio Biotech Co. (Hangzhou, China). Antibody against p-PKD1 was purchased from Proteintech Biotech Co. (Wuhan, China). The other chemicals and reagents used were of analytical grade available.

### Network pharmacology analysis

#### Prediction of drug targets for CRT

The Similarity Ensemble Approach (SEA; https://sea.bkslab.org/), Super-PRED database (Super-PRED; https://prediction.charite.de/index.php), Pharmmapper database (PharmMapper; http://lilab-ecust.cn/pharmmapper/), and Swiss Target Prediction (Swiss; http://www.swisstargetprediction.ch/) were utilized to obtain the CRT targets (Liu et al., 2023; Wang K. et al., 2024), which were further matched to their corresponding standardized gene names using Universal Protein Resource database (UniProt; https://www.uniprot.org/), with species being set as “*Home sapiens*”. Subsequently, the duplicate targets from the databases above were removed, and the final CRT targets were obtained.

#### Prediction of targets for obesity

*Homo sapiens* targets associated with obesity were screened from the GeneCards (Li T. et al., 2024) (https://www.genecards.org/) human gene database using “obesity” as the search term.

#### Construction of the protein-protein interaction (PPI) network, kyoto encyclopedia of genes and genomes (KEGG) enrichment and gene ontology (GO) analyses

The CRT-associated targets were compared with the obesity-associated targets to identify common targets. The potential protein targets obtained were imported into STRING (https://string-db.org/) to establish a PPI network (Zhou et al., 2024; Huang et al., 2024b). To ensure data reliability, the protein-protein interactions were filtered further with a minimum combined score of 0.9 (highest confidence), selecting hide disconnected nodes in the network in the network display option (He et al., 2024). The PPI network was further calculated, and hub genes were identified and visually analyzed using Cytoscape 3.10.3 software.

To analyze the potential function of the identified proteins, the DAVID database (https://david.ncifcrf.gov/summary.jsp) was adopted for GO analysis, including biological process (BP), cellular component (CC), and molecular function (MF), as well as KEGG enrichment analysis (Cheng et al., 2024; Hu et al., 2023). The complete *H. sapiens* gene dataset was used as the background for the analysis, with a significance criterion set at p < 0.05.

### Cell culture and differentiation

3T3-L1 preadipocytes were obtained from ATCC (Manassas, VA, USA) and maintained in DMEM supplemented with 10% FBS and 1% penicillin-streptomycin at 37 °C in a humidified incubator with 5% CO_2_. For differentiation, cells were seeded in 6-well plates and grown to ∼80% confluence. Two days post-confluence (Day 0), differentiation was induced with an induction medium containing 0.5 mM IBMX, 1 μM Dex, and 10 μg/mL insulin (described as MDI) in 10% FBS-DMEM. After 48 h (Day 2), the medium was replaced with DMEM containing 10% FBS and 10 μg/mL insulin for an additional 2 days, followed by maintenance in DMEM with 10% FBS until Day 6 (Lee et al., 2022). By Day 6, cells exhibited robust lipid accumulation and the appearance of visible lipid droplets, indicative of successful adipogenic differentiation.

### Drug treatments

Different CRT treatment protocols were applied across experiments based on specific objectives, with detailed administration procedures provided in the respective figures. CRT was dissolved in 0.1% DMSO (v/v), diluted in culture medium, and administered to 3T3-L1 cells at various stages of differentiation according to the experimental design. To evaluate whether the AMPK pathway was involved in the effect of CRT, cells were co-treated with AMPK inhibitor Compound C (Zhang et al., 2023) (10 μM) and CRT for 48 h. This co-treatment was performed during the early phase of adipocyte differentiation (Day 0-Day 2).

### Cell viability

Cell viability was assessed using the MTT assay. Briefly, 3T3-L1 preadipocytes were seeded in 96-well plates at a density of 1 × 10^4^ cells per well and allowed to attach overnight. Cells were then treated with various concentrations of CRT (0, 0.001, 0.003, 0.01, 0.03, 0.1, 0.3, 1 μM) for 48 h or 6 days. At the end of the treatment period, 20 µL of MTT solution (5 mg/mL in PBS) was added to each well and incubated at 37 °C for 4 h. The medium was carefully removed, and 150 µL of DMSO was added to dissolve the formazan crystals (Wang W. Z. et al., 2024). Absorbance was measured at 570 nm using a microplate reader.

### Oil Red O staining and lipid quantification

On Day 6, cells were washed with phosphate-buffered saline (PBS), fixed with 4% paraformaldehyde for 30 min at room temperature, and stained with Oil Red O (0.5% in isopropanol) for 30 min. Excess stain was removed, and cells were washed with distilled water. Stained lipid droplets were visualized under a light microscope (Chu et al., 2022). For quantification, Oil Red O was extracted using 100% isopropanol, and absorbance was measured at 500 nm.

### Quantitative real-time PCR (qRT-PCR)

Total RNA was extracted using the TRIzol reagent (Invitrogen, USA) according to the manufacturer’s instructions. RNA concentration and purity were determined using a NanoDrop 2000 spectrophotometer (Thermo Fisher Scientific, USA), with samples showing an A260/A280 ratio between 1.8 and 2.0 considered acceptable (Wu et al., 2024; Fleige and Pfaffl, 2006). For cDNA synthesis, 1 μg of total RNA was reverse transcribed using the PrimeScript™ RT Reagent Kit (Takara, Japan) following the manufacturer’s protocol. The reverse transcription reaction was carried out in a final volume of 20 μL under the following conditions: 37 °C for 15 min, followed by 85 °C for 5 s to inactivate the reverse transcriptase. Synthesized cDNA was stored at −20 °C until further use. All reactions were performed using a Bio-Rad T100 Thermal Cycler (Bio-Rad, USA) (Fleige and Pfaffl, 2006; Li M. R. et al., 2024). The relative mRNA expression of other genes was measured by qRT‒PCR using SYBR Green Master Mix (Vazyme Biotech Co., Nanjing, China). Relative mRNA expression levels of genes were normalized to *Gapdh* using the 2^−ΔΔCT^ method. The primer sequences are detailed in Table 1.

**TABLE 1:** Primers sequences.

Primer for	Primer sequence 5′ to 3′
*Pparg*	Forward: CGA​GTC​TGT​GGG​GAT​AAA​GC
	Reverse: CCG​GCA​GTT​AAG​ATC​ACA​CC
*Cebpa*	Forward: GGT​GGA​CAA​GAA​CAG​CAA​CG
	Reverse: CGT​TGT​TTG​GCT​TTA​TCT​CG
*Fasn*	Forward: GGA​GGT​GGT​GAT​AGC​CGG​TAT
	Reverse: TGG​GTA​ATC​CAT​AGA​GCC​CAG
*Srebf1*	Forward: AGG​TCA​CCG​TTT​CTT​TGT​GG
	Reverse: AGT​TCA​ACG​CTC​GCT​CTA​GG
*Fabp4*	Forward: AAG​GTG​AAG​AGC​ATC​ATA​ACC​CT
	Reverse: TCA​CGC​CTT​TCA​TAA​CAC​ATT​CC
*Klf5*	Forward: CCG​GAG​ACG​ATC​TGA​AAC​ACG
	Reverse: GTT​GAT​GCT​GTA​AGG​TAT​GCC​T
*Gapdh*	Forward: GGT​GAA​GGT​CGG​TGT​GAA​CG
	Reverse: CTC​GCT​CCT​GGA​AGA​TGG​TG

### Western blotting assay

Protein lysates from cells were prepared according to standard protocols (Luo et al., 2024). Total protein concentrations were determined using a BCA protein assay kit (Beyotime, China) following the manufacturer’s instructions. The absorbance was measured at 562 nm using a microplate reader (Thermo Fisher Scientific, USA) to quantify protein content. Equal amounts of protein were separated by 10% sodium dodecyl sulfate-polyacrylamide gel electrophoresis (SDS-PAGE) by electrophoresis at 80 V for stacking and 120 V for resolving. Proteins were then transferred to the PVDF membranes (Millipore, Burlington, MA, USA), which were pre-activated in methanol for 1 min, using a wet transfer system at 300 mA for 90 min at 4 °C. Membranes were blocked with 5% bovine serum albumin (BSA, Beyotime) in TBST (Tris-buffered saline containing 0.1% Tween-20) for 1 h at room temperature (Luo et al., 2025), followed by overnight incubation at 4 °C for approximately 16 h with primary antibodies against C/EBPα, PPARγ, Adiponectin, p-AMPK, AMPK, p-ACC, ACC, and GAPDH. Primary antibodies were diluted in 5% (w/v) BSA prepared in TBST. After washing three times with TBST, then, membranes were incubated with HRP-conjugated secondary antibodies for 1 h at room temperature. Protein bands were detected using an ultra-sensitive ECL chemiluminescence kit (Beyotime) on a chemiluminescence imaging system (Tanon 5,200, Yuanpinghao Biotechnology, Beijing, China) (Luo et al., 2023). Densitometric analysis of band intensities was performed using ImageJ software.

### Statistical analysis

Data are presented as the means ± SEM. Statistical analysis was performed with one-way analysis of variance (ANOVA) using GraphPad Prism. *P* values less than 0.05 (*P* < 0.05) were considered statistically significant.

## Results

### Network pharmacology prediction

**FIGURE 2:**
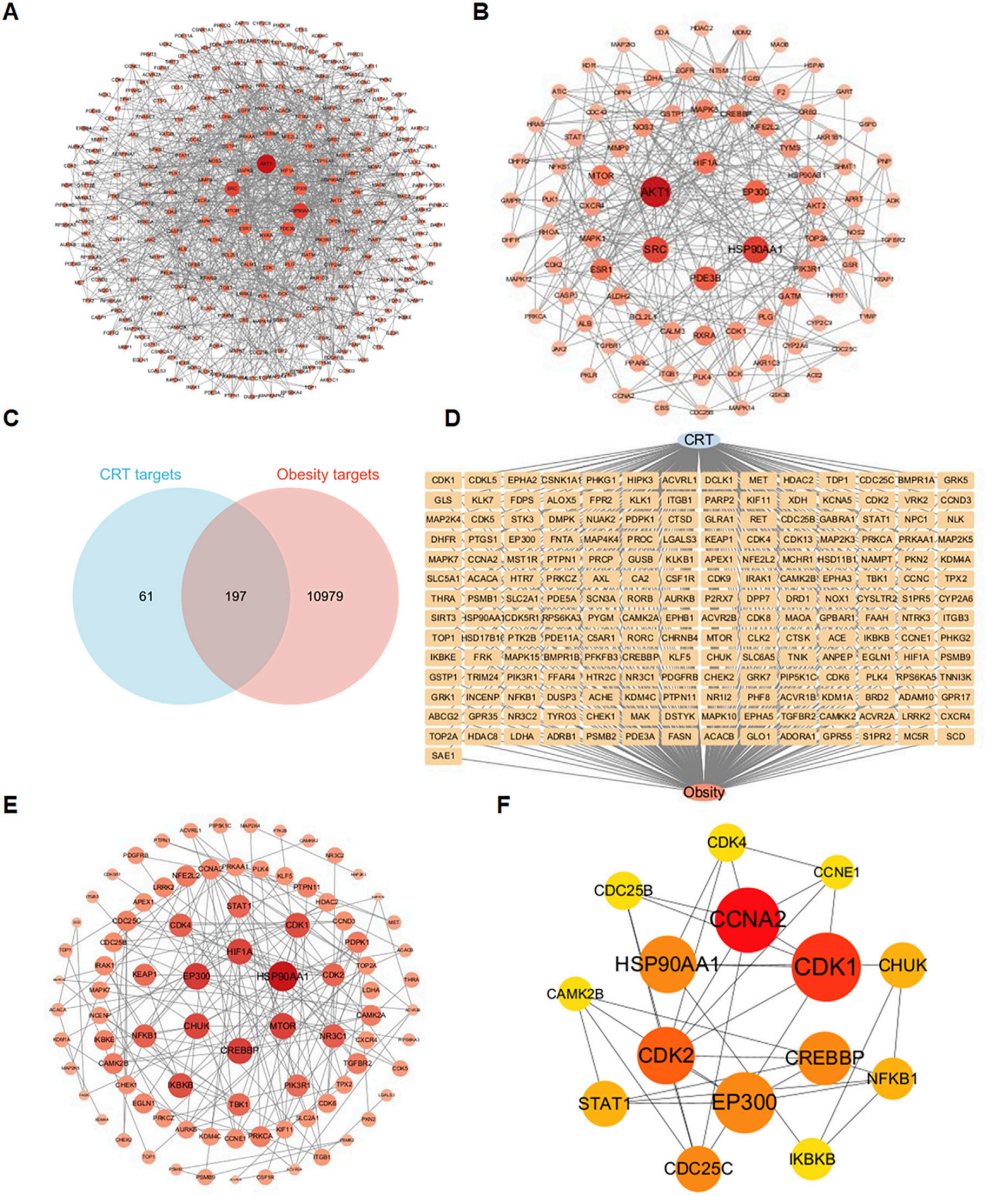
Identification of intersection targets shared by CRT and obesity. **(A)** A PPI network of targets regulated by CRT. There were 289 nodes and 3,116 edges in the network. **(B)** The 88 core targets of CRT with average BC, closeness centrality, and degree values higher than the average. **(C,D)** 197 intersection targets of CRT and obesity. **(E)** The PPI network of CRT and obesity intersection targets. **(F)** Key targets of CRT for treating obesity based on cytoHubba analysis.

A total of 258 targets related to CRT were found through the overlapping of data from the Swiss Target Prediction, Similarity Ensemble Approach, SuperPred, and PharmMapper Server databases. Additionally, by searching the GeneCards database, a total of 11176 targets associated with obesity were identified. A comparison of the CRT-associated targets with the obesity-associated targets led to the identification of 197 common targets. A total of 289 nodes and 3,116 edges were found in the PPI network utilizing a minimum combined score of 0.9, as shown in Figure 2A. These targets were screened respectively according to degree value, betweenness centrality (BC), and closeness centrality. There were 88 targets with average BC, closeness centrality, and degree values higher than the average, as shown in Figure 2B.

Degree values higher than the average, as shown in Figure 2B. Based on the Venn diagram, 197 common targets (Figure 2C) were screened, and the diagram of a CRT-obesity-targets network (Figure 2D) was utilized using Cytoscape software. A total of 87 nodes and 174 edges were found in the PPI network utilizing a minimum combined score of 0.9, as shown in Figure 2E. These targets were filtered respectively according to cytoHubba analysis, 15 core targets were identified and ranked by degree values.

**FIGURE 3:**
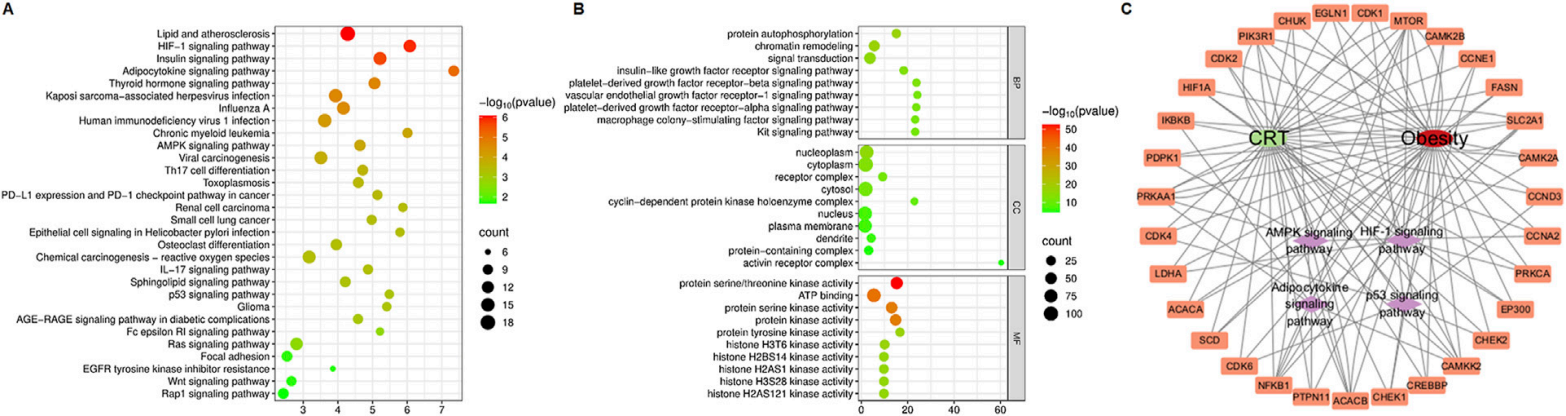
KEGG, GO analysis, and drug-target-pathway-disease network construction. **(A)** The dot plot of KEGG-enriched pathways. **(B)** The enriched BP, CC, and MF terms from GO analysis were displayed in bubble charts. **(C)** The drug-target-pathway-disease network was constructed by Cytoscape3.10.3.

To investigate the molecular mechanism of the anti-obesity effects of CRT, 197 interaction targets were utilized to perform KEGG and GO enrichment analyses via the DAVID database. The KEGG enrichment analysis revealed that the HIF-1 signaling pathway, adipocytokine signaling pathway, and AMPK signaling pathway are associated with the anti-obesity effect of CRT (Figure 3A). Furthermore, GO gene function enrichment analysis identified 81 biological processes, indicating that CRT primarily participates in protein phosphorylation, protein autophosphorylation, and chromatin remodeling. The cellular components mainly focused on the nucleoplasm, cytoplasm, and receptor complex. The effects of CRT on molecular functions mainly included protein serine/threonine kinase activity, ATP binding, and protein serine kinase activity (Figure 3B). Moreover, the drug-target-pathway-disease network intuitively displayed the potential targets of CRT in treating obesity enriched in the HIF-1 signaling pathway, adipocytokine signaling pathway, AMPK signaling pathway, and p53 signaling pathway (Figure 3C). CRT may significantly contribute to the anti-obesity impact via modulating adipogenesis, as indicated by network pharmacology.

### Impact of CRT on the differentiation of 3T3-L1 preadipocytes

**FIGURE 4:**
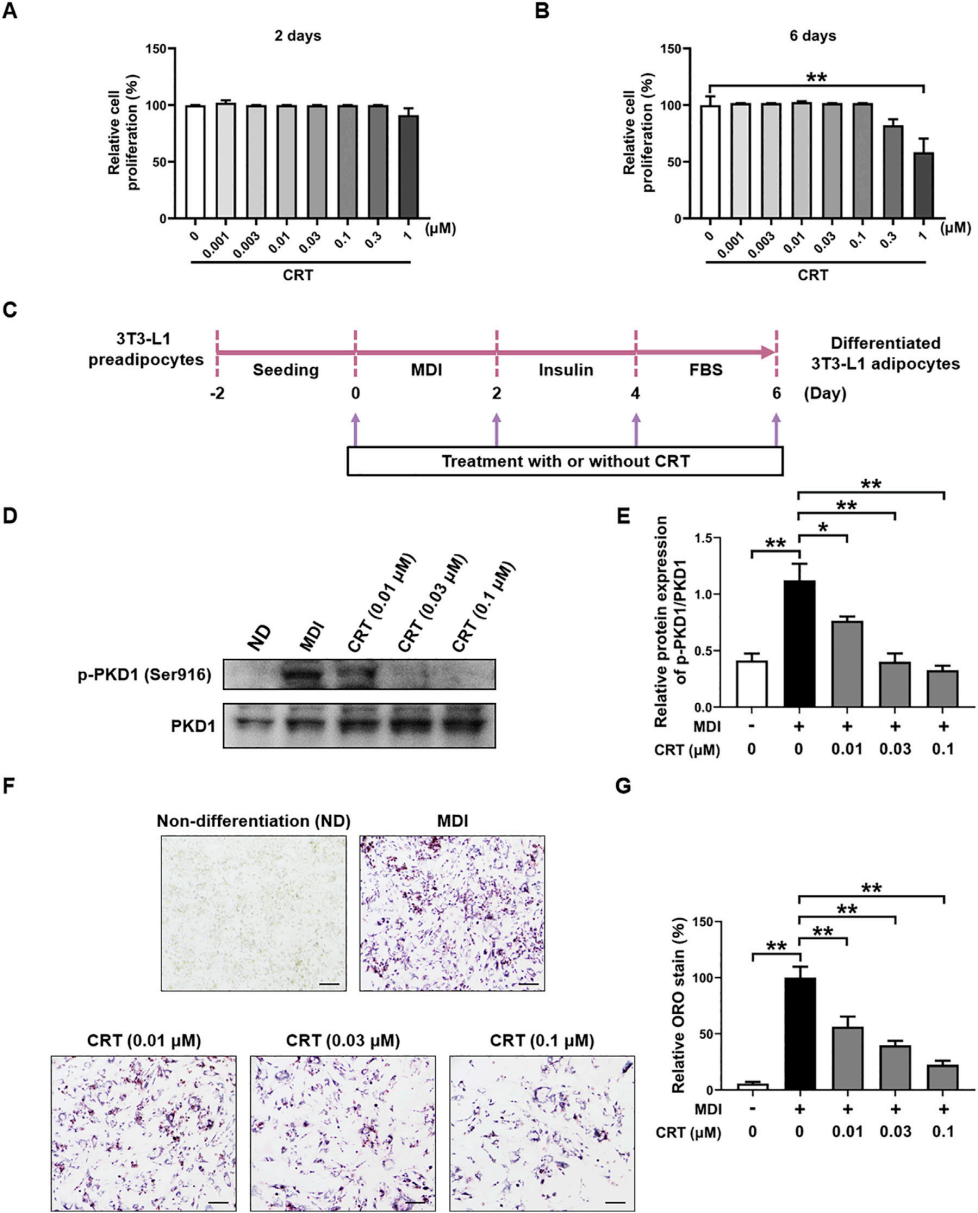
Effects of CRT on the differentiation of 3T3-L1 preadipocytes. **(A,B)** Cell viability analysis was used to determine the total number of living cells in 3T3-L1 preadipocytes treated with different concentrations of CRT for 2 days or 6 days. **(C)** Scheme of 3T3-L1 preadipocyte differentiation experimental design. **(D)** On Day 6, total protein was extracted. Expression levels of p-PKD1 and PKD1 were analyzed by Western blotting. **(E)** Quantitative analysis of individual protein levels, results were presented as mean ± SEM (n = 3). **(F,G)** 3T3-L1 preadipocytes were induced to differentiate with an induction medium containing MDI and FBS in the presence or absence of different concentrations of CRT up to 6 days of differentiation. Relative lipid accumulation stained by Oil red O was visualized in optical microscopy (×20 magnification) and quantified by measuring absorbance at 500 nm. Scale bar: 100 µm. The data are presented as the mean ± SEM (n = 3). **P < 0.01, *P < 0.05.

To evaluate the effect of CRT on cell viability during adipocyte differentiation, the MTT assay was performed, with cells treated with CRT at concentrations up to 1 μM for either 48 h or 6 days. Our results demonstrated that CRT did not significantly affect cell viability at concentrations up to 0.3 μM at both time points. (Figures 4A,B). Based on these findings, concentrations ranging from 0.01 to 0.1 μM were chosen for further experiments to investigate the effects of CRT on adipocyte differentiation.

Differentiation of 3T3-L1 preadipocytes in the presence of a differentiation medium containing MDI resulted in significant lipid accumulation (Mubtasim et al., 2023). To evaluate the effects of CRT on adipocyte differentiation, 3T3-L1 preadipocytes were treated with CRT (0.01, 0.03, 0.1 μM) for a duration of 6 days (Figure 4C). Western blot analysis revealed that PKD1 phosphorylation was significantly increased upon MDI-induced differentiation, indicating the activation of PKD1 during adipocyte differentiation. Notably, treatment with CRT markedly reduced p-PKD1 levels without affecting total PKD1 expression, indicating that CRT effectively suppresses PKD1 activation (Figures 4D,E). Lipid accumulation was assessed on Day 6 using Oil Red O staining. In comparison to the MDI group, CRT treatment significantly diminished lipid droplet formation in a dose-dependent manner, indicating a strong inhibitory effect on adipocyte differentiation (Figure 4F). Quantitative analysis of the extracted Oil Red O further confirmed these results, showing a significant reduction in lipid content with increasing concentrations of CRT (Figure 4G). These findings suggest that CRT hinders lipid droplet production during 3T3-L1 adipocyte differentiation.

### CRT inhibits adipogenesis in the early stage of differentiation

**FIGURE 5:**
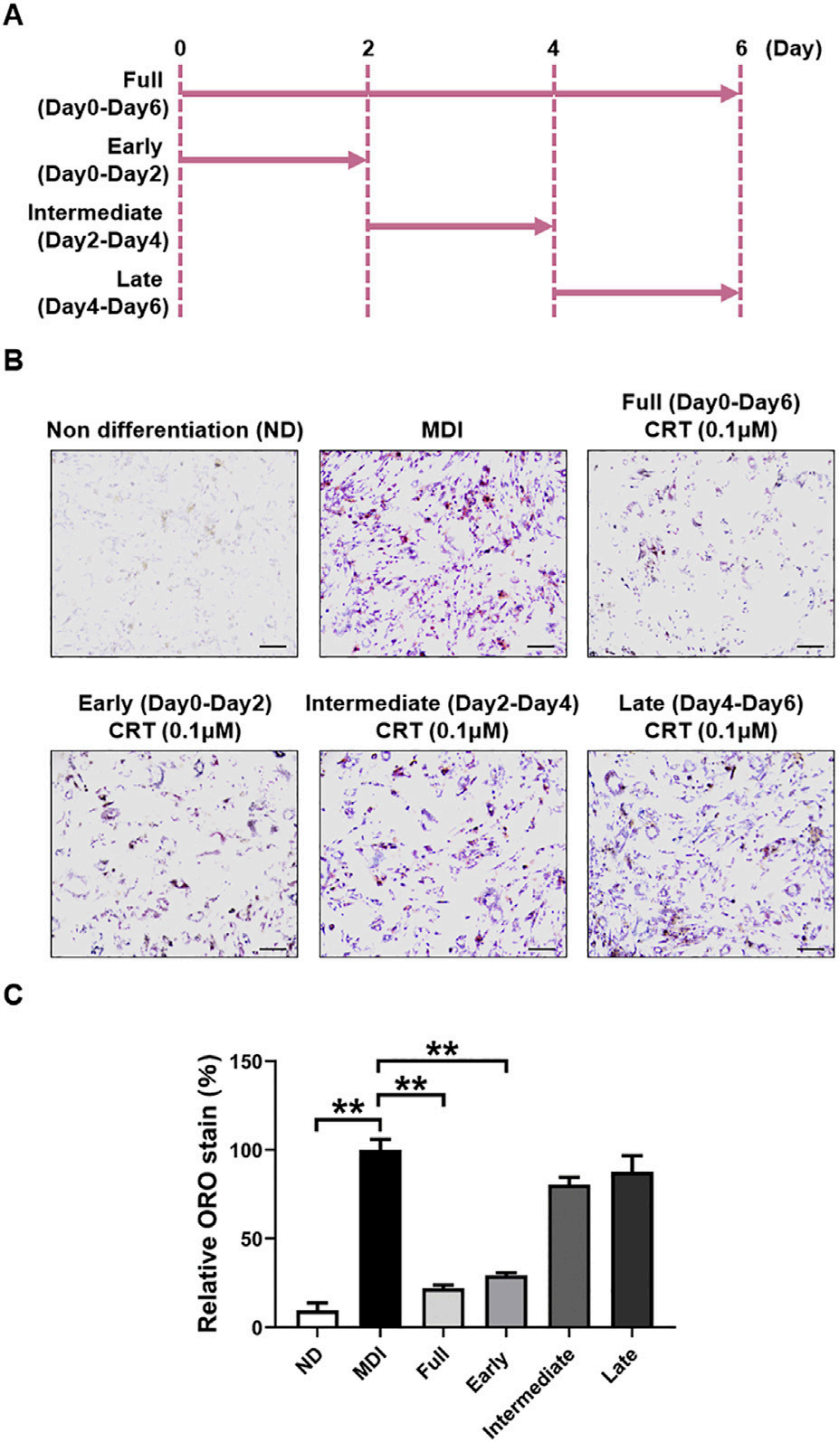
CRT mostly inhibits the early stage of adipocyte differentiation. **(A)** Time schedule for CRT treatment during 3T3-L1 cell differentiation. **(B)** Representative images of Oil red O staining of 3T3-L1 adipocytes treated with CRT at different stages of differentiation (×20 magnification). Scale bar: 100 µm. **(C)** Relative lipid accumulation stained by Oil red O was measured by a microplate reader at 500 nm. The data are presented as the mean ± SEM (n = 3). **P < 0.01.

To assess whether CRT exerts its effect during a specific phase of adipocyte differentiation, 3T3-L1 preadipocytes were treated with CRT (0.1 μM) during distinct stages: early (Days 0–2), intermediate (Days 2–4), and late (Days 4–6) (Figure 5A). Lipid accumulation was evaluated by Oil Red O staining on Day 6. Compared to MDI-treated control cells, 6-day treatment with CRT significantly reduced intracellular lipid droplets by approximately 78%. Among the different stages, treatment during the early phase resulted in the most substantial inhibition of lipid accumulation, with reductions of approximately 71%, 20%, and 12% for the early, intermediate, and late stages, respectively (Figures 5B,C). These findings suggest that CRT most effectively suppresses adipogenesis when administered during the early stage of differentiation.

### Effect of CRT on the expressions of differentiation-associated genes

**FIGURE 6:**
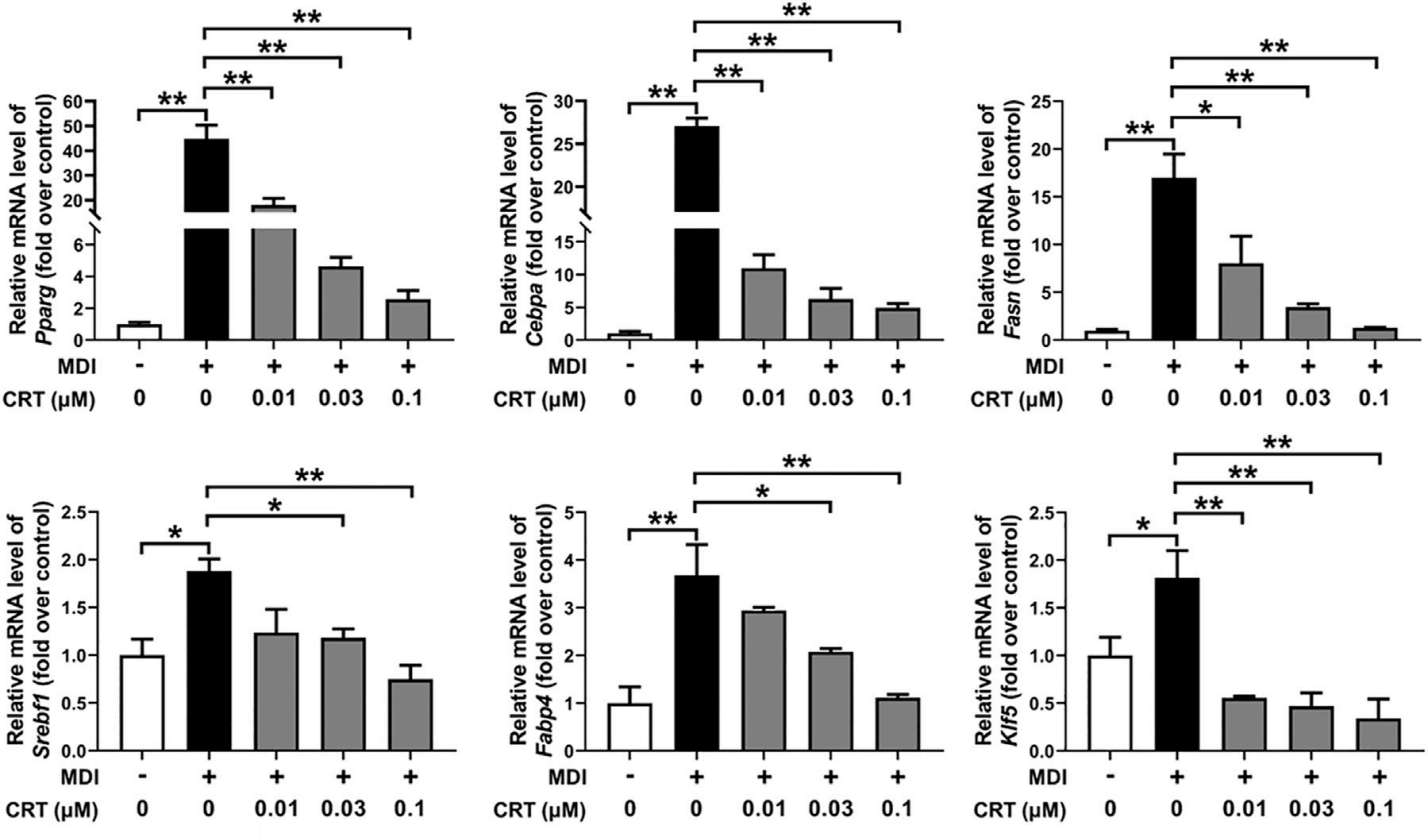
Effect of CRT on the adipocyte differentiation-associated genes. 3T3-L1 preadipocytes were induced to differentiate with an induction medium containing MDI and 10% FBS in the presence or absence of different concentrations of CRT during the early stage (Days 0–2). On Day 6, total RNA was extracted, and the mRNA levels of key adipocyte differentiation-associated genes (*Pparg*, *Cebpa*, *Fasn*, *Srebf1*, *Fabp4*, and *Klf5*) were analyzed by RT-qPCR. Gene expression was normalized to *Gapdh*, and data are presented as fold change relative to the control. The data are presented as the mean ± SEM (n = 3). **P < 0.01; *P < 0.05.

To investigate the molecular mechanisms by which CRT suppresses adipogenesis, the mRNA expression levels of essential adipogenic transcription factors and markers were assessed by qRT-PCR. 3T3-L1 preadipocytes were treated with CRT (0.01, 0.03, and 0.1 μM) during the early stage of differentiation (Days 0–2), and total RNA was extracted on Day 6. CRT treatment resulted in a significant, dose-dependent downregulation of *Pparg, Cebpa, Fasn, Srebf1, Fabp4*, and *Klf5* compared to the MDI group (Figure 6). These genes are critical regulators of adipocyte differentiation and lipid metabolism.

### Effects of CRT on the expression of key adipogenic markers

**FIGURE 7:**
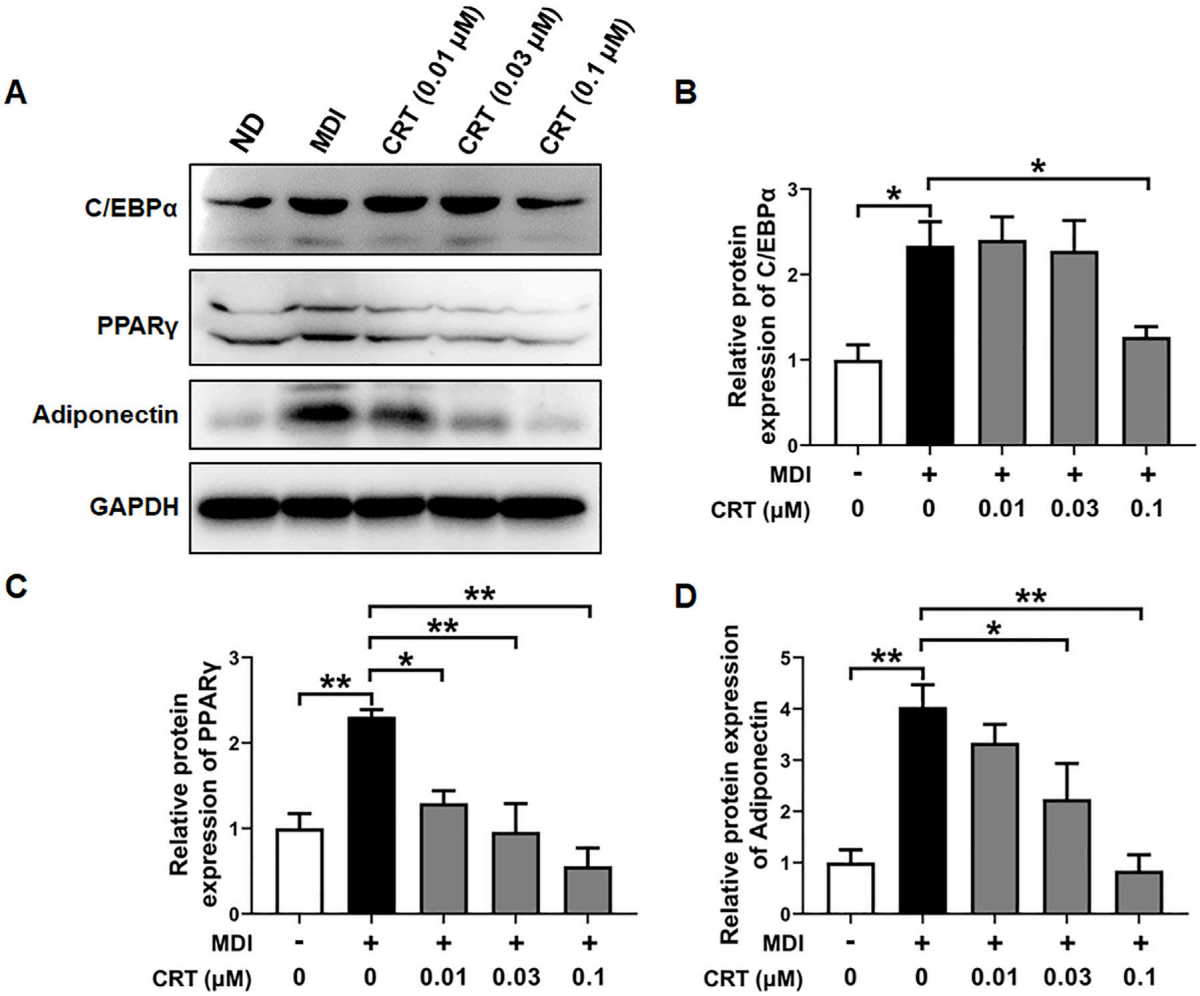
Effect of CRT on the adipocyte differentiation-associated proteins. 3T3-L1 preadipocytes were induced to differentiate with an induction medium containing MDI and 10% FBS in the presence or absence of different concentrations of CRT during the early stage (Days 0–2). On Day 6, total protein was extracted. **(A)** Expression levels of C/EBPα, PPARγ, Adiponectin, and GAPDH were analyzed by Western blotting. **(B–D)** Quantitative analysis of individual protein levels, results were presented as mean ± SEM (n = 3). **P < 0.01; *P < 0.05.

To further evaluate the inhibitory effects of CRT on adipocyte differentiation at the protein level, the expression of key adipogenic markers—C/EBPα, PPARγ, and Adiponectin (Patel et al., 2024; Lee et al., 2022)—was assessed by Western blotting. 3T3-L1 preadipocytes were treated with CRT (0.01, 0.03, and 0.1 μM) during the early stage of differentiation (Days 0–2), and protein samples were collected on Day 6. The results showed that CRT treatment significantly decreased the protein expression of C/EBPα, PPARγ, and Adiponectin in a dose-dependent manner compared to the MDI-treated group (Figures 7A–D).

### Effects of CRT on the AMPK signaling pathway in 3T3-L1 cells

**FIGURE 8:**
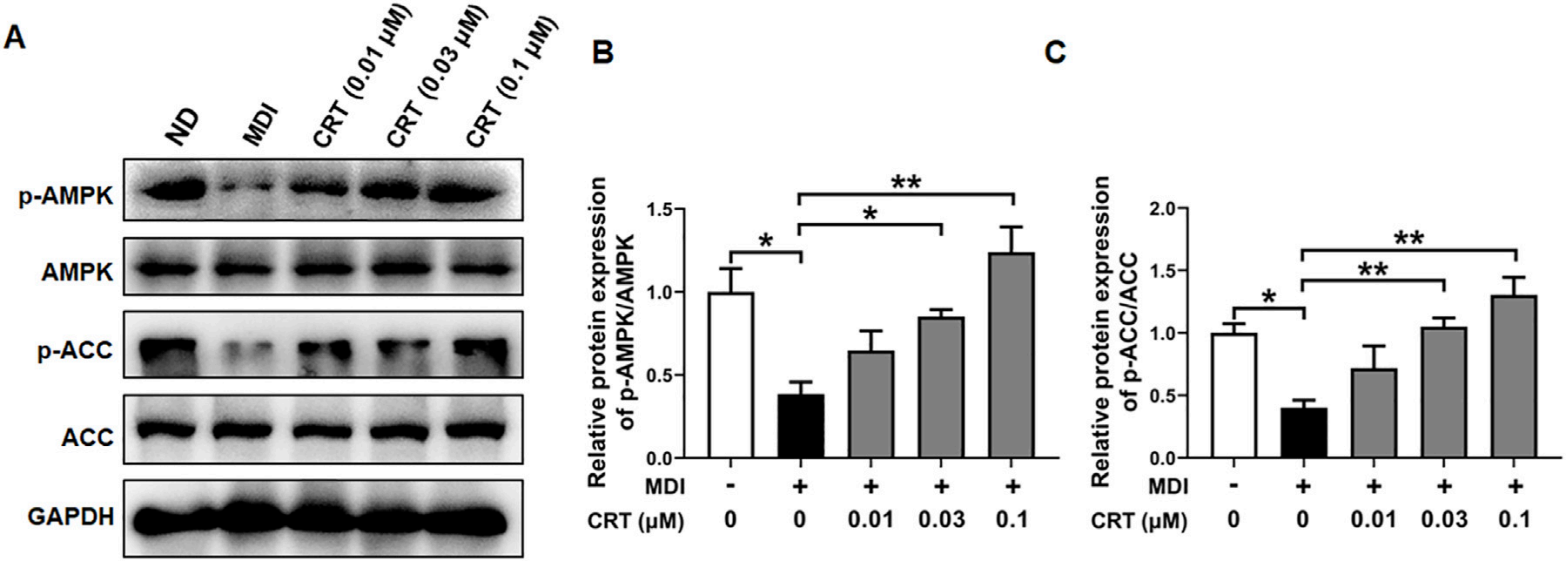
CRT Activates AMPK Signaling in 3T3-L1 Adipocytes. 3T3-L1 preadipocytes were treated with different concentrations of CRT during the early stage of differentiation (Days 0–2). On Day 6, total protein was extracted. **(A)** The expression levels of phosphorylated AMPK (p-AMPK), total AMPK, phosphorylated acetyl-CoA carboxylase (p-ACC), and total ACC were analyzed by Western blotting. GAPDH was used as a loading control. **(B,C)** Quantitative analysis of individual protein levels, results were presented as mean ± SEM (n = 3). **P < 0.01; *P < 0.05.

To gain further insight into the mechanism underlying CRT-mediated inhibition of adipocyte differentiation, we examined the activation status of the AMPK signaling pathway, which is pivotal in regulating energy homeostasis and adipocyte differentiation. Western blotting analysis was conducted to assess the phosphorylation levels of AMPK and its downstream target ACC. The CRT treatment significantly elevated the phosphorylation of both AMPK and ACC in 3T3-L1 cells compared to the MDI-induced group, although the total protein levels of AMPK and ACC remained unchanged (Figures 8A–C). These results suggest that CRT activates the AMPK signaling pathway, thereby contributing to the suppression of adipocyte differentiation.

### CRT inhibited adipocyte differentiation via the AMPK pathway

**FIGURE 9:**
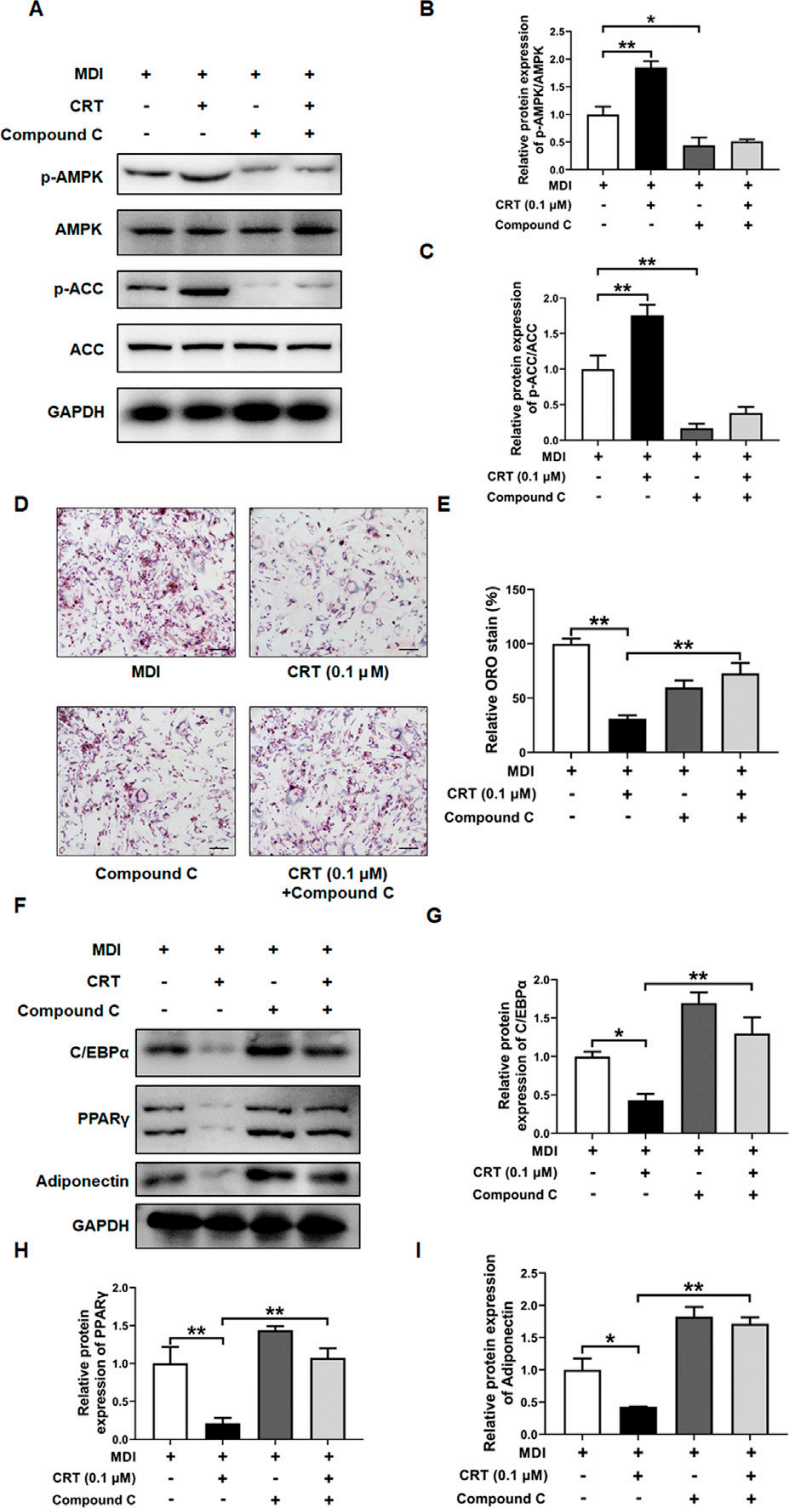
AMPK inhibitor (Compound C) attenuates the inhibitory effect of CRT on adipocyte differentiation. 3T3-L1 preadipocytes were treated with CRT (0.1 μM) in the presence or absence of Compound C (10 μM) during the early stage of differentiation (Days 0–2). on Day 6, cells were harvested for analysis. **(A)** Total protein was extracted, and the expression levels of phosphorylated AMPK (p-AMPK), total AMPK, phosphorylated acetyl-CoA carboxylase (p-ACC), and total ACC were analyzed by Western blotting. GAPDH was used as a loading control. Representative blot images are shown. **(B,C)** Quantitative analysis of individual protein levels, results were presented as mean ± SEM (n = 3). **(D)** Representative images of Oil Red O-stained adipocytes under different treatments (×20 magnification), Scale bar: 100 µm. **(E)** Relative lipid accumulation stained by Oil red O was measured by a microplate reader at 500 nm. Data are presented as a percentage of the control (mean ± SEM, n = 3). **(F)** Expression levels of C/EBPα, PPARγ, Adiponectin, and GAPDH were analyzed by Western blotting **(G–I)** Quantitative analysis of individual protein levels, results were presented as mean ± SEM (n = 3). **P < 0.01; *P < 0.05.

To determine whether CRT suppresses adipocyte differentiation through the AMPK signaling pathway, 3T3-L1 preadipocytes were treated with CRT (0.1 μM) alone or in combination with Compound C (10 μM), a selective AMPK inhibitor, during the early stage of differentiation (Days 0–2). Western blot analysis revealed that CRT significantly increased the phosphorylation of AMPK and its downstream target ACC, indicating activation of the AMPK pathway. However, co-treatment with Compound C effectively suppressed CRT-induced phosphorylation of both AMPK and ACC (Figures 9A–C). Consistently, Oil Red O staining on Day 6 demonstrated that CRT markedly reduced lipid accumulation, while the inhibitory effect was significantly attenuated by Compound C (Figures 9D,E). Moreover, the downregulation of key adipogenic markers—C/EBPα, PPARγ, and Adiponectin—observed with CRT treatment was also reversed upon Compound C co-treatment (Figures 9F–I). These findings suggest that CRT inhibits adipocyte differentiation at least in part through AMPK pathway activation, highlighting AMPK as a critical mediator of its anti-adipogenic effects.

## Discussion

As a key contributor to fat mass accumulation, adipogenesis represents a promising therapeutic target for obesity (Wan et al., 2023). In the present study, we demonstrated that CRT, a pan inhibitor of PKD, suppresses adipocyte differentiation in 3T3-L1 preadipocytes. This inhibitory effect is accompanied by reduced lipid accumulation, downregulation of adipogenic markers (such as PPARγ and C/EBPα), and activation of the AMPK signaling pathway. Furthermore, pharmacological inhibition of AMPK with Compound C partially reversed the anti-adipogenic effects of CRT, suggesting that AMPK activation plays a crucial role in mediating the observed phenotype. These findings provided new insights into the role of PKD in adipocyte biology and identified CRT as a candidate for anti-obesity intervention.

While CRT has been studied for its anti-proliferative and anti-inflammatory effects in various cancer models through inhibition of PKD isoforms (Harikumar et al., 2010; Li et al., 2018; Cui et al., 2023), its role in adipocyte biology remains largely unexplored. Our study is the first to demonstrate that pharmacological inhibition of PKD by CRT disrupts adipogenic differentiation, highlighting a previously unrecognized role for PKD signaling in adipose development. A study by Mona et al. demonstrated that adipocyte-specific deletion of PKD1 in mice enhances energy expenditure and protects against diet-induced obesity (Löffler et al., 2018). Similarly, PKD2 was shown to promote dietary lipid absorption in the intestine by facilitating chylomicron formation and secretion (Trujillo-Viera et al., 2021). Our findings further supported the metabolic relevance of PKD and suggested that its inhibition may favor an anti-adipogenic state. Notably, as a pan-inhibitor of PKD, CRT raises the possibility that its anti-adipogenic effects may stem from the inhibition of multiple nodes in the PKD signaling axis. This is consistent with previous research demonstrating that modulation of kinase signaling pathways can profoundly influence adipogenic outcomes. For instance, inhibition of mTORC1, ERK, or JAK-STAT pathways similarly reduces adipogenesis by attenuating transcriptional cascades (Zhang et al., 2024; Guo et al., 2024). Our findings extend this paradigm to PKD, an understudied kinase involved in adipocyte differentiation.

The 3T3-L1 preadipocyte model remains a gold standard for studying adipogenesis due to its reproducibility and physiological relevance (Al-Sayegh et al., 2021). Adipogenesis in 3T3-L1 cells is a stepwise process involving growth arrest, mitotic clonal expansion, early-stage differentiation, and terminal differentiation (Zebisch et al., 2012; Choi et al., 2020). Each phase is tightly regulated by a cascade of transcription factors and signaling pathways. Among these stages, the early phase of differentiation is particularly critical, as it involves the induction of C/EBPβ and C/EBPδ, which subsequently activate C/EBPα and its binding partner PPARγ—the two master regulators of terminal adipogenesis (Mubtasim et al., 2023; Wang et al., 2022). These transcription factors, once upregulated, drive the expression of multiple lipogenic genes, including Fasn, Fabp4/aP2, and Srebf1 (Choi et al., 2020; Lee et al., 2022). CRT treatment dramatically reduces protein and mRNA levels of C/EBPα and PPARγ, demonstrating an extreme inhibitory effect on the core transcriptional machinery driving adipogenesis. Notably, CRT most effectively reduced lipid accumulation when applied during early differentiation (days 0–2), suggesting it primarily targets early-stage regulatory events in adipogenesis. Further supporting this notion, CRT treatment also downregulated Klf5, a transcription factor essential for the commitment and early differentiation of preadipocytes (Lee et al., 2022). This aligns with previous reports that early-phase regulators are essential for triggering the terminal differentiation cascade. For example, Klf5 knockout impairs early adipogenic gene expression and precludes full adipocyte maturation (Oishi et al., 2005). In addition, CRT significantly downregulated the mRNA expression of Fabp4, Fasn, and Srebf1, which are important downstream targets of PPARγ and C/EBPα for lipid synthesis and storage (Choi et al., 2020; Lee et al., 2022; Wang et al., 2022). The dose-dependent suppression of these genes further suggests that CRT disrupts the transcriptional network essential for adipocyte maturation and lipid accumulation. Collectively, our findings suggest that CRT hinders adipogenesis predominantly at the early stage by disrupting the activation of the PPARγ-C/EBPα axis and subsequently suppressing the expression of lipogenic genes necessary for adipocyte maturation and lipid accumulation.

AMPK is an essential energy sensor activated under cellular energy stress, functioning to restore energy homeostasis by shifting metabolism away from anabolic and toward catabolic pathways (Zhou et al., 2023; Göransson et al., 2023; Moseti et al., 2016; Wang et al., 2025). During adipocyte differentiation, AMPK activation typically acts as a negative regulator (Zhou et al., 2023; Göransson et al., 2023). This occurs through phosphorylation of its catalytic α-subunit, which inhibits ATP-consuming anabolic processes and promotes ATP-generating catabolic pathways (Zhou et al., 2023; Göransson et al., 2023). The resulting energy shift suppresses key adipogenic transcription factors, such as PPARγ and C/EBPα, and downregulates lipogenic enzymes like FASN and ACC, thus obstructing the progression from preadipocytes to mature adipocytes (Göransson et al., 2023). Due to its broad role in metabolic regulation, the AMPK pathway represents a promising therapeutic target for obesity and related metabolic disorders. Consistent with network pharmacology predictions, our study found that CRT activates AMPK, strongly implicating this pathway in the anti-adipogenic mechanism of CRT. This notably connects CRT with AMPK activators, despite its original creation for distinct purposes. This also suggests that PKD inhibition could indirectly activate AMPK, a mechanistic link that has not yet been fully elucidated. Whether this connection occurs through altered upstream kinase activity or is due to cellular energy stress remains unknown and warrants further investigation.

Collectively, our data demonstrated that CRT suppresses adipocyte differentiation in 3T3-L1 cells by activating the AMPK signaling pathway, leading to downregulation of PPARγ and C/EBPα and reduced expression of key lipogenic genes. These findings establish CRT as a novel pharmacological candidate for targeting adipogenesis, hence expanding its therapeutic applicability beyond oncology. Considering AMPK’s pivotal function in energy metabolism, insulin sensitivity, and hepatic lipid regulation, CRT may offer multifaceted benefits for metabolic syndrome. However, additional *in vivo* studies are necessary to validate its efficacy, evaluate bioavailability and safety, and investigate tissue-specific effects related to obesity and insulin resistance. Additionally, future research should investigate molecular targets beyond the AMPK pathway to gain a more comprehensive understanding of the underlying mechanisms. Extending treatment durations, evaluating effects during late differentiation stages and in fully mature adipocytes, alongside broader target profiling and translational studies, will be necessary to strengthen the clinical relevance and therapeutic potential of CRT.

## Data Availability

The original contributions presented in the study are included in the article/supplementary material, further inquiries can be directed to the corresponding author.
